# Chemical profiling of *Staurogyne stenophylla* via UPLC-HRMS and *in silico* investigation into its anti-inflammatory potential

**DOI:** 10.3389/fvets.2026.1805430

**Published:** 2026-06-11

**Authors:** Mengping Zhang, Ziwen Wang, Shini Lin, Yongqian Liu, Zihao Zhang, Xiujuan Xiao, Guohua Yu, Yanhui Liu, Qiong Wu

**Affiliations:** 1 School of Life Sciences, Longyan University, Longyan, Fujian, China; 2 School of Life Sciences, Fujian Agriculture and Forestry University, Fuzhou, Fujian, China

**Keywords:** active components, anti-inflammatory, network pharmacology, *Staurogyne stenophylla*, UPLC-HRMS

## Abstract

*Staurogyne stenophylla* (Hemsl.) C. B. Clarke ex S. Moore (*S. stenophylla*) has long been recognized in traditional folk medicine for its anti-inflammatory properties. However, its specific bioactive components and underlying molecular mechanisms remain unclear, which limits its scientific application as a novel anti-inflammatory feed additive for livestock and poultry. This study employed ultra-performance liquid chromatography-high-resolution mass spectrometry (UPLC-HRMS) to characterize the chemical profile of the *S. stenophylla* aqueous extract. A network pharmacology framework was established by retrieving inflammation-related targets from GeneCards and OMIM databases and identifying intersecting targets with the active components. Protein–protein interaction (PPI) networks were constructed via STRING and Cytoscape. Gene ontology (GO) and Kyoto Encyclopedia of genes and genomes (KEGG) enrichment analyses were performed using DAVID. Interactions between core components and targets were verified by CB-DOCK2 molecular docking and Gromacs molecular dynamics (MD) simulations. UPLC-HRMS identified 1,720 chemical components, which were narrowed down to 15 main active components and 72 inflammation-associated intersecting targets. The PPI network highlighted 16 core targets, including SRC, PPARG, TP53, PTGS2, and CASP3. Enrichment analyses indicated that these targets are primarily involved in the NF-κB and MAPK signaling pathways. Molecular docking demonstrated strong binding affinities (< −6.0 kcal/Mol) between 7 core components (e.g., morin, Glabridin, apigenin) and 5 key targets. Notably, MD simulations revealed that the Glabridin-PTGS2 complex formed a distinct stable conformation with superior stability. This study elucidates that *S. stenophylla* exerts anti-inflammatory effects through a multi-component, multi-target mechanism, primarily by modulating the NF-κB and MAPK pathways. These findings provide a theoretical basis and scientific support for developing *S. stenophylla* as a functional feed additive to improve health in livestock and poultry farming.

## 1 Introduction

Inflammatory diseases represent a pervasive challenge in the livestock and poultry industries. These conditions not only directly compromise animal health and welfare but also impair growth performance and can lead to large-scale mortality, resulting in severe economic losses (1, 2). Effective prevention and control of these inflammatory diseases depend on comprehensive management strategies. Primarily, this requires establishing robust hygiene practices and strict biosecurity measures, alongside the implementation of appropriate vaccination protocols tailored to specific farm conditions. Furthermore, it is critical to mitigate potential predisposing factors that induce inflammation, such as pathogen infections, environmental stress, and malnutrition. Moreover, the early detection of diseases combined with prompt and appropriate therapeutic interventions is essential for effective animal management (3–5).

Respiratory and intestinal inflammation are particularly prevalent in veterinary practice. Respiratory inflammation is primarily triggered by pathogens that activate the NF-κB and MAPK signaling pathways, leading to the release of proinflammatory cytokines (e.g., IL-1β, IL-6, and TNF-*α*), which subsequently causes pulmonary inflammation and tissue damage (6, 7). Similarly, intestinal inflammation is typically induced by pathogens via the TLR4/MyD88/NF-κB/MAPK pathway, resulting in impaired intestinal barrier function and immune cell infiltration (8, 9).

Currently, the livestock industry relies heavily on a combination of prophylactic medication and therapeutic interventions to manage these disorders, predominantly utilizing tetracyclines, β-lactam antibiotics, and immunomodulators (10, 11). However, the overuse of antibiotics accelerates the emergence of bacterial resistance, disrupts intestinal microbiota homeostasis, and inhibits beneficial commensal bacteria, thereby weakening animal immunity (12, 13). Furthermore, antibiotic residues in animal-derived products pose significant “One Health” risks, including potential organ damage (e.g., hepatotoxicity and nephrotoxicity) and allergic reactions in human consumers (12, 14). Consequently, there is an urgent need to develop anti-inflammatory therapies based on traditional Chinese medicines (TCMs), which are characterized by significant antimicrobial efficacy, favorable safety profiles, and a low risk of residue accumulation.

As documented in the *Flora of China*, *Staurogyne stenophylla* (Hemsl.) C. B. Clarke ex S. Moore (*S. stenophylla*), locally known as Baibeicao, is a perennial herb belonging to the class Magnoliopsida, order Lamiales, genus Staurogyne (family Acanthaceae) (15). In traditional Chinese folk medicine, this plant has been historically employed to treat various inflammatory conditions, including gastroenteritis, tonsillitis, nephritis, chronic hepatitis, and conjunctivitis. These ethnomedicinal applications suggest that *S. stenophylla* possesses significant potential in anti-inflammatory therapies, offering a novel perspective for its application in veterinary anti-inflammatory strategies.

Despite its empirical use, the specific chemical profile of *S. stenophylla* remains largely undefined, and the molecular mechanisms and therapeutic targets underlying its effects are poorly understood. This gap highlights the necessity for a comprehensive investigation into its pharmacological properties. Ultra-performance liquid chromatography-high-resolution mass spectrometry (UPLC-HRMS) couples ultra-performance liquid chromatography with high-resolution mass spectrometry. Its advantages—including high sensitivity, high resolution, rapid analysis, and multi-dimensional data acquisition—make it a robust tool for identifying the chemical constituents of *S. stenophylla* and establishing quality control standards (16). Furthermore, network pharmacology, which leverages bioinformatics and network analysis, allows for the prediction of complex interactions between bioactive components and biological targets. This holistic approach assists in deciphering the multi-target mechanisms of herbal medicines (17–19). In addition, protein–protein interaction (PPI) networks can reveal key targets and core modules, opening new avenues for multi-target therapeutic strategies, which can be subsequently validated by molecular docking and molecular dynamics simulations (20). The integration of these technologies advances the modernization of TCM research by providing a rationale for drug development (21). Therefore, a combined approach using UPLC-HRMS, network pharmacology, and molecular dynamics is essential to systematically explore the value of *S. stenophylla*.

In this study, UPLC-HRMS was employed to comprehensively identify the chemical constituents of the aqueous extract of *S. stenophylla*. Integrating this data with network pharmacology, we constructed “component-target,” “component-target-disease,” and PPI networks to map the inflammatory targets associated with these active ingredients. Gene Ontology (GO) and Kyoto Encyclopedia of Genes and Genomes (KEGG) enrichment analyses were performed to elucidate the biological functions and signaling pathways involved. Finally, molecular docking was utilized to validate the binding affinity between candidate bioactive components and key therapeutic targets. Collectively, this study provides a scientific basis for elucidating the pharmacological material basis and molecular mechanisms underlying the anti-inflammatory activity of *S. stenophylla*.

## 2 Materials and methods

### 2.1 Plant materials and reagents

*Staurogyne stenophylla* (Hemsl.) C. B. Clarke ex S. Moore was collected from the forests of Shangchi Village, Zhongchi Town, Wuping County, Fujian Province, China. LC–MS grade methanol and acetonitrile were purchased from Fisher Chemical (Waltham, MA, USA). LC–MS grade formic acid was obtained from Honeywell (Charlotte, NC, USA). Ultrapure water was purchased from Millipore (Burlington, MA, USA).

### 2.2 Main instruments

The analysis was performed using a Q-Exactive HFX mass spectrometer coupled with a Vanquish Ultra-Performance Liquid Chromatography (UHPLC) system (Thermo Fisher Scientific, Bremen, Germany). Other equipment included a 5,430 R low-temperature high-speed centrifuge (Eppendorf, Hamburg, Germany), an ME104 electronic balance (Mettler Toledo, Shanghai, China), a QT-1 vortex mixer (Xiexu Medical, Shanghai, China), an FD-1C-80 freeze dryer (Shanghai Bilon Instruments Co., Ltd., Shanghai, China), and an SB-4200D ultrasonic cleaner (Ningbo Scientz Biotechnology Co., Ltd., Ningbo, China).

### 2.3 Sample preparation and extraction

The fresh aerial parts of *S. stenophylla* were harvested from the wild and dried in a constant-temperature oven at 60 °C for 12 h. A 100 g portion of the dried sample was accurately weighed and transferred into a ceramic decocting vessel. For the initial extraction, 1,000 mL of distilled water was added and boiled, and 500 mL of the filtrate was collected. The remaining residue was subjected to two additional decoctions with appropriate volumes of distilled water. All resulting filtrates were pooled to a total volume of 1,000 mL. The combined filtrate was subsequently concentrated to a final volume of 100 mL using a rotary evaporator. The obtained aqueous extract was then utilized for chemical profiling via UPLC-HRMS.

### 2.4 Chromatographic conditions

Chromatographic separation was achieved on a Waters ACQUITY UPLC HSS T3 column (2.1 mm X 100 mm, 1.8 μm). The column temperature was maintained at 35 °C, and the flow rate was set at 0.3 mL/min. The mobile phase consisted of 0.1% formic acid in water (A) and 0.1% formic acid in acetonitrile (B). The gradient elution program is detailed in Table 1.

**Table 1:** Ultra-performance liquid chromatography gradient elution program.

Time (min)	Mobile phase A (%)	Mobile phase B (%)
Initial	95	5
3.0	75	25
8.5	55	45
14.0	5	95
17.0	2	98
17.2	95	5
20.0	95	5

### 2.5 Mass spectrometry conditions

MS and MS/MS data acquisition was performed using the Q-Exactive HFX mass spectrometer equipped with a heated electrospray ionization (HESI) source operating in both positive and negative ion modes. The key parameters were set as follows: spray voltage, 3.8 kV (positive) and 3.5 kV (negative); sheath gas flow rate, 45 arb; auxiliary gas flow rate, 20 arb; capillary temperature, 320 °C; and auxiliary gas heater temperature, 350 °C. The instrument operated in Full-MS/dd-MS2 mode. The resolution was set at 60,000 for Full MS (MS1) and 15,000 for MS2. The top 10 most abundant precursor ions were selected for fragmentation with stepped normalized collision energies (NCE) of 20, 40, and 60. The mass scanning range was *m/z* 90–1,300.

### 2.6 Data analysis workflow

The raw mass spectrometry data were converted to an accessible format using ProteoWizard software. Subsequently, peak alignment, retention time correction, and peak extraction were performed using the XCMS package. Metabolite annotation was conducted by matching the acquired MS and MS/MS data against a proprietary high-resolution mass spectrometry database for Traditional Chinese Medicine (TCM). The criteria for putative identification were established with a mass tolerance of <25 ppm for precursor ions and a fragmentation matching score of >0.7, where a higher score signifies greater spectral similarity. According to the Metabolomics Standards Initiative (MSI) guidelines, the identification of these metabolites corresponds to Level 2 (putatively annotated compounds), as the annotation relies on accurate mass and MS/MS spectral library matching without confirmation by authentic reference standards. To evaluate the technical reproducibility and stability of the detection system, Pearson correlation analysis was performed on five replicate injections of the sample. Relative quantification of the identified compounds was achieved based on their average peak areas. In accordance with standard practices in untargeted metabolomics, no external standard curves were employed, and the relative abundance was used for downstream comparative analysis.

### 2.7 Network pharmacology research

#### 2.7.1 Screening of active components and target retrieval for *S. stenophylla*

The chemical components of *S. stenophylla* identified via UPLC-HRMS were subjected to a dual-screening strategy based on their relative abundances and the Traditional Chinese Medicine Systems Pharmacology Database and Analysis Platform (TCMSP).^1^ Initially, the identified compounds were cross-referenced with the TCMSP database to select components with potential biological targets. To ensure data reliability, the analysis was performed in five replicates. The average peak area and relative standard deviation (RSD) were calculated for each compound, and only those exhibiting high reproducibility were retained. The relative content of each component was determined using the following formula:


$$\text{Relative content}\;(\%)=(\begin{array}{l} \text{average peak area of the compound}\\ /\text{total average peak area of}\;\mathrm{all}\;\\ \text{matched compounds} \end{array})\times 100\%.$$

The components were then ranked in descending order of their relative content. By integrating this ranking with previously reported anti-inflammatory bioactivities in the literature, the top 15 compounds were selected as representative active components. Subsequently, the UniProt database^2^ was utilized to normalize the target protein information and convert the identified target gene names into Official Gene Symbols.

#### 2.7.2 Construction of component-target and drug-inflammation networks

The 15 screened active components and their corresponding binding targets were imported into Cytoscape software (v3.7.1) to construct a preliminary “Component-Target” interaction network. Concurrently, inflammation-related genes were retrieved from the GeneCards^3^ and OMIM^4^ databases using the keyword “inflammation.” Recognizing that these databases are predominantly derived from human clinical data, manual curation was conducted to ensure their applicability and relevance to veterinary research. Genes previously documented to be involved in the regulation of inflammation in poultry, or those highly conserved across species, were prioritized for inclusion. Entries lacking confirmed physiological associations with animal inflammation were strictly excluded. After the removal of duplicates, a core dataset of inflammation-related targets was established.

Subsequently, intersection analysis between the component-specific targets and the core inflammation-related targets was performed using the Venny online tool^5^ to identify the intersecting gene sets. Finally, the active components, intersecting targets, and the target disease (inflammation) were integrated into Cytoscape to construct a multidimensional “Drug-Component-Target-Inflammation” network. Through the topological layout of nodes and edges, this network visually elucidates the holistic regulatory mechanisms by which the active compounds modulate inflammatory processes via specific targets.

#### 2.7.3 PPI network analysis, core target screening, and functional enrichment

The intersecting targets were mapped into the STRING database^6^ with the confidence score set to 0.4 to obtain protein–protein interaction (PPI) data. Subsequently, the CytoNCA plugin in Cytoscape was utilized to calculate the topological parameters of each node, including Degree Centrality (DC), Betweenness Centrality (BC), Closeness Centrality (CC), and Average Shortest Path Length (ASPL). To ensure objectivity in the screening process, crucial core targets within the network were identified based on the median distribution characteristics of the topological parameters (DC > 4, BC > 14.70, CC > 0.10). Following this, Gene Ontology (GO) functional annotation and Kyoto Encyclopedia of Genes and Genomes (KEGG) pathway enrichment analyses of the intersecting targets were conducted using the DAVID database.^7^ Enrichment terms significantly associated with anti-inflammatory effects (*p* < 0.05) were selected. Data visualization was subsequently performed using an online bioinformatics platform^8^ to elucidate the potential biological functions and key signaling pathways mediating the anti-inflammatory effects of the active components.

#### 2.7.4 Molecular docking and molecular dynamics (MD) simulation

Based on the results of the PPI topological analysis, the top five core targets and seven representative core active components (screened based on their relative UPLC abundance and literature-reported anti-inflammatory activities) were selected for molecular docking. The 2D structures of the active components were obtained from the PubChem database,^9^ while the 3D crystal structures of core proteins were retrieved from the RCSB Protein Data Bank^10^ (PDB). Docking was performed using the CB-DOCK2 (22) server (), where the binding pose with the lowest binding energy was considered the optimal conformation. The docking results showed that the PTGS2-Glabridin complex exhibited the lowest binding energy (−10.1 kcal/mol), demonstrating the strongest affinity and binding stability among all combinations. Therefore, it was selected as a representative model for molecular dynamics (MD) simulation using GROMACS v2023.02. The GAFF force field was applied to the ligand, while the Amber99sb-ILDN force field and TIP3P water model were used for the protein and solvent, respectively. The system was equilibrated with 100 ps NVT and 100 ps NPT phases, followed by a 100 ns production run based on the docked structure. To evaluate the binding stability and dynamic behavior of the complex, trajectory analysis included calculations of root mean square deviation (RMSD), root mean square fluctuation (RMSF), radius of gyration (Rg), solvent-accessible surface area (SASA), and hydrogen bonds (H-bonds). Additionally, the free energy landscape (FEL) was constructed in 2D and 3D based on the dynamic variations of RMSD and Rg.

## 3 Results

### 3.1 Analysis of chemical components in the aqueous extract of *S. stenophylla*

**Figure 1:**
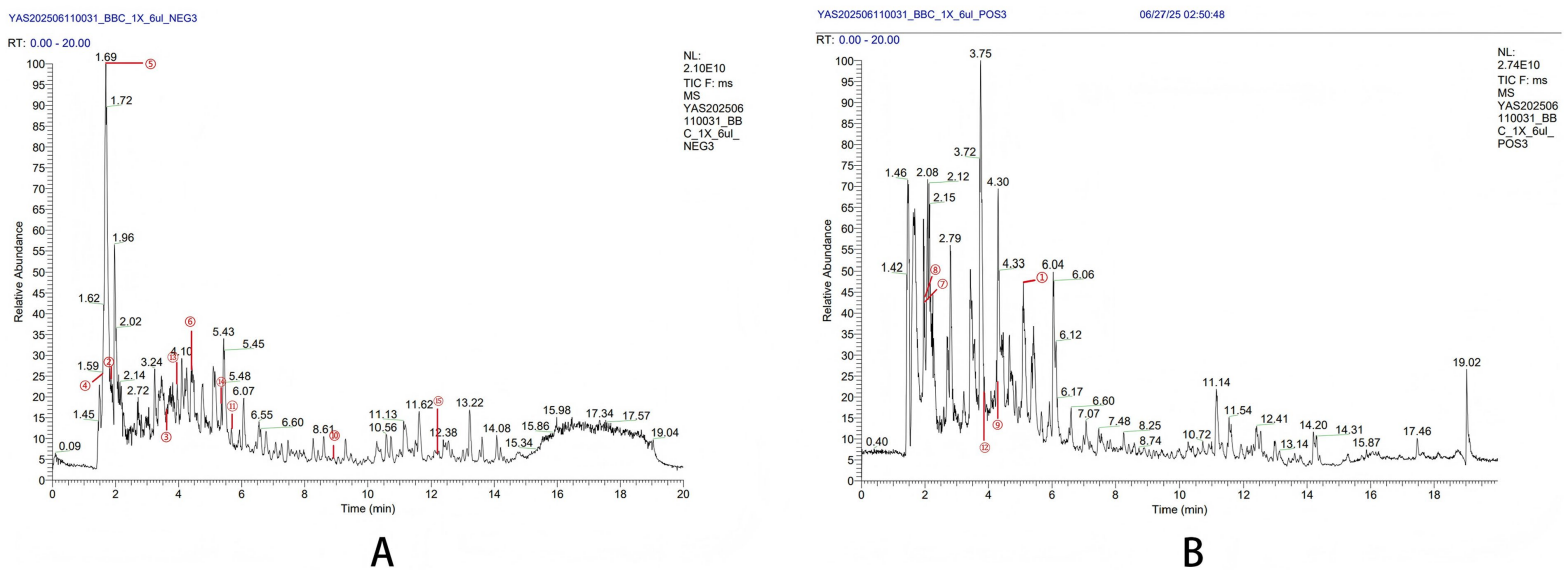
Total ion current (TIC) chromatograms of aqueous extract of *S. stenophylla* in negative **(A)** and positive **(B)** ion modes.

**Figure 2:**
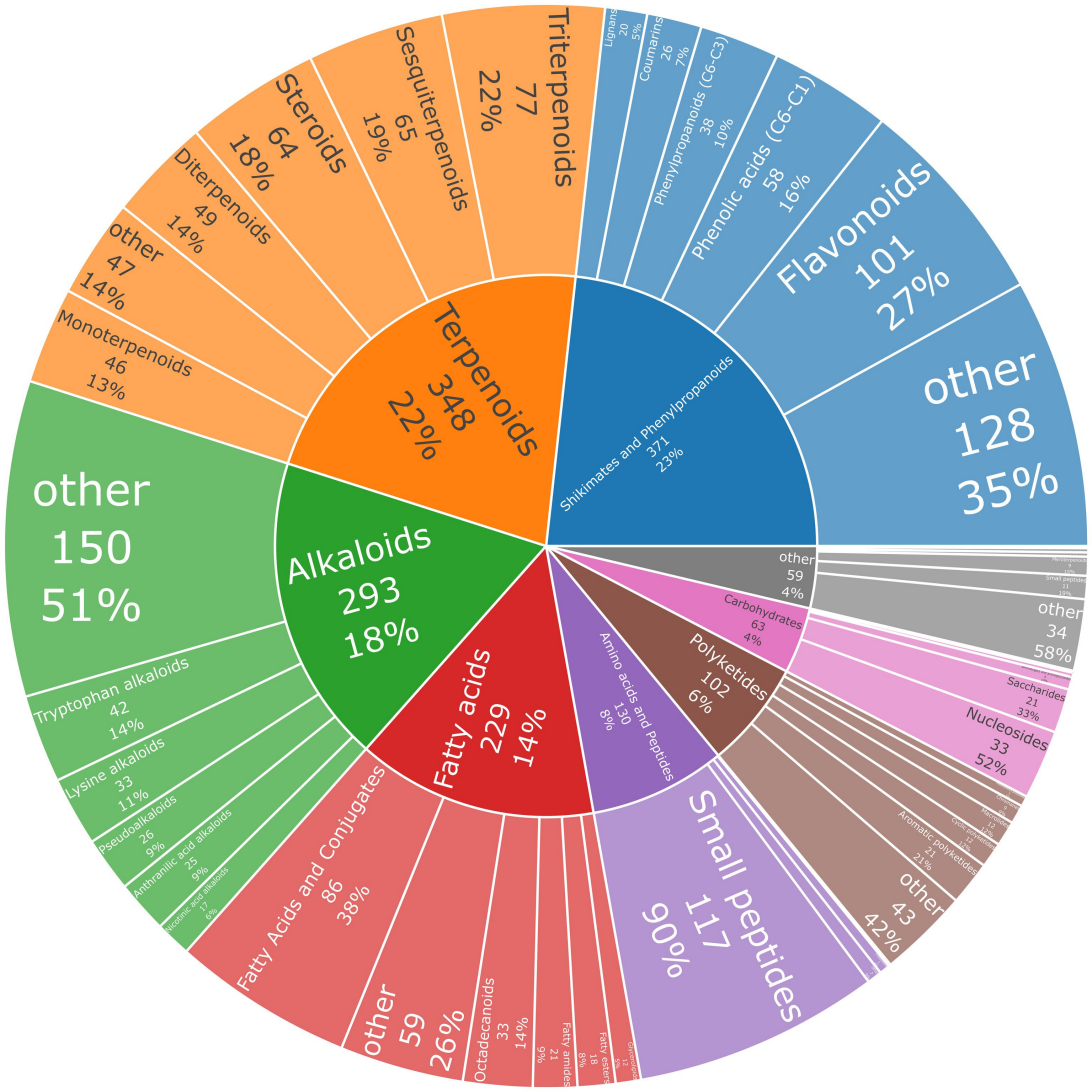
Pie chart showing the proportional distribution of chemical classes in *S. stenophylla*.

UPLC-HRMS was employed to analyze the chemical components of the aqueous extract of *S. stenophylla.* A total of 1,720 active components were putatively identified in both positive and negative ion modes (Table 2), and the corresponding total ion chromatograms (TIC) were obtained (Figure 1). The secondary fragmentation spectrum matching scores for all compounds were above 0.7, indicating high reliability of the identification results. The identified compounds were categorically annotated using the NPClassifier method; the proportion of identified compounds across various pathways and major superclasses is illustrated in Figure 2. The results indicated that the compounds could be classified into seven major categories with the following proportions: Shikimates and Phenylpropanoids (23%), Terpenoids (22%), Alkaloids (18%), Fatty acids (14%), Amino acids and Peptides (8%), Polyketides (6%), and Carbohydrates (4%). The remaining 4% of compounds were not classified into these aforementioned categories. The Pearson correlation coefficients for the data from five replicate injections of the sample were all greater than 0.9, demonstrating excellent detection reproducibility and confirming that the data are stable and reliable.

**Table 2:** Statistics of compounds identified in positive and negative ion modes.

Detection ion mode	Number of identified substances
Positive ion mode (POS)	1,118
Negative ion mode (NEG)	646
Total of positive and negative ion modes	1,720

Total of positive and negative ion modes: compounds identified as the same in both positive and negative ion modes are not counted repeatedly.

### 3.2 Network pharmacology prediction analysis

#### 3.2.1 Screening of active components and targets of *S. stenophylla*

Based on the compound information of *S. stenophylla* obtained via UPLC-HRMS analysis, combined with their inclusion status in the TCMSP database and relative content parameters, 15 representative components (Table 3) were screened from the 1,720 initially identified compounds (Supplementary Table 1) to serve as potential active components for subsequent network pharmacology analysis. These 15 components were then imported into the TCMSP database for target prediction to collect the potential targets corresponding to each component. After removing duplicate entries, a total of 75 unique potential targets were ultimately obtained.

**Table 3:** Identification of components in the aqueous extract of *S. stenophylla.*

Components	Retention time tR/min	Formula	Theoretical value		Measured valuem/z	Error (mg/kg)
			[M + H]+	[M + H]-		
Morin	4.61	C15H10O7	303.0499		303.0500	0.4
Glabridin	3.95	C20H20O4		323.1347	323.1289	18
Succinic acid	1.82	C4H6O4		117.0173	117.0182	7.6
Vanillic acid	3.64	C8H8O4		167.0322	167.0330	4.5
Stachyose	1.58	C24H42O21		665.2147	665.2152	0.8
Citric acid	1.69	C6H8O7		191.0189	191.0195	2.9
Caffeate	4.41	C9H8O4		179.0339	179.0344	3
Uracil	1.99	C4H4N2O2	113.0346		113.0349	2.4
4-Guanidinobutyric acid	1.97	C5H11N3O2	146.0922		146.0924	1.1
Esculetin	4.28	C9H6O4	179.0339		179.0340	0.7
Apigenin	8.9	C15H10O5		269.0456	269.0457	0.2
Naringin	5.68	C27H32O14		579.1714	579.1724	1.8
Vanillin acetate	3.83	C10H10O4	195.0652		195.0653	0.4
Hirsutrin	5.36	C21H20O12		463.0881	463.0883	0.4
[6]-Gingerol	12.19	C17H26O4		293.1759	293.176	0.3

#### 3.2.2 Component-target network

**Figure 3:**
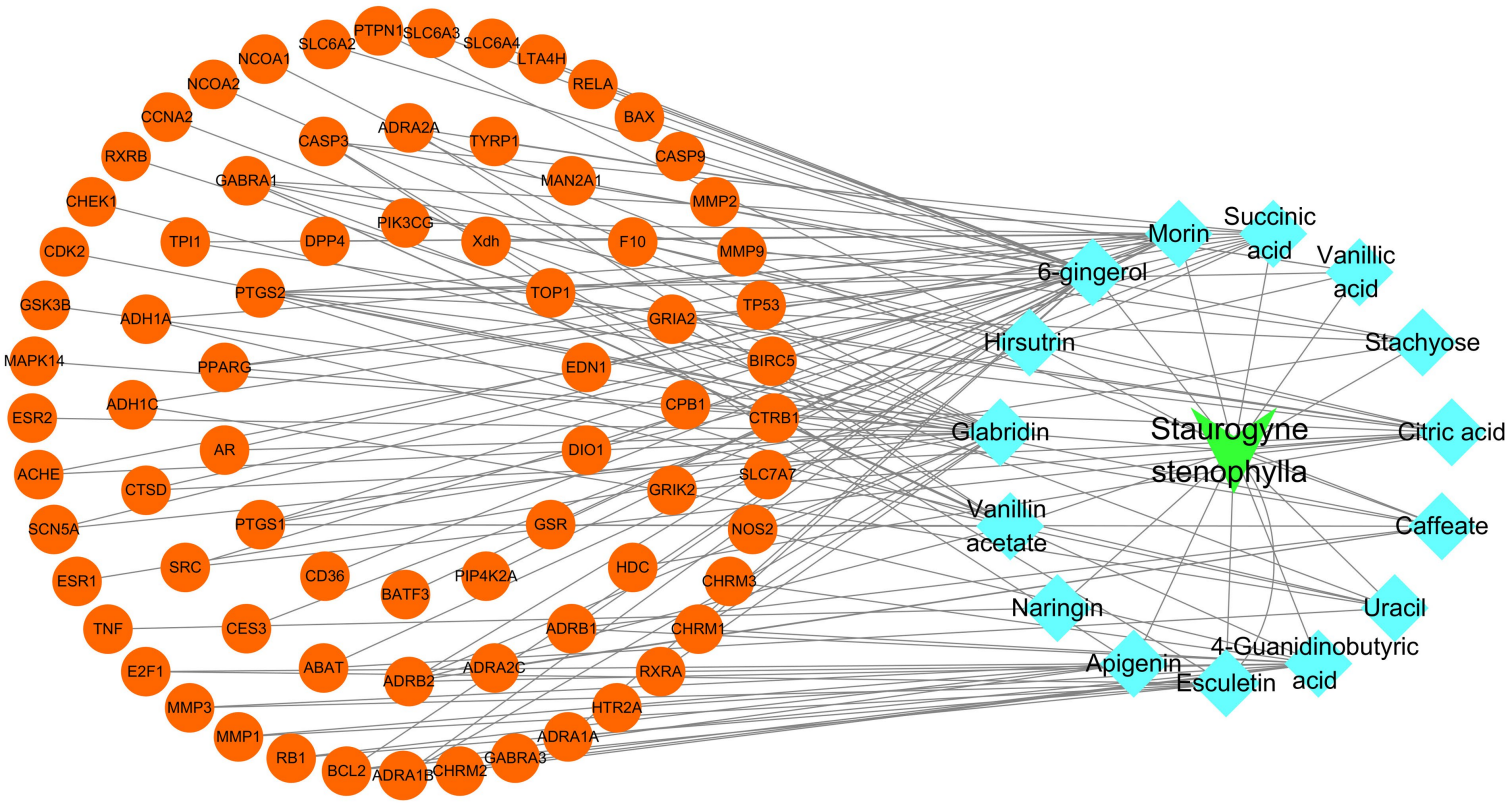
Active component-target network of *S. stenophylla*.

The 15 active components and their corresponding 75 targets were imported into Cytoscape software to construct a component-target visualization network. The network consists of 1 herbal medicine, 15 active components, 91 nodes, and 145 edges (Figure 3). A higher degree value indicates that a node is connected to more nodes, reflecting a more significant regulatory role within the entire network.

#### 3.2.3 Acquisition of inflammation-related targets

**Figure 4:**
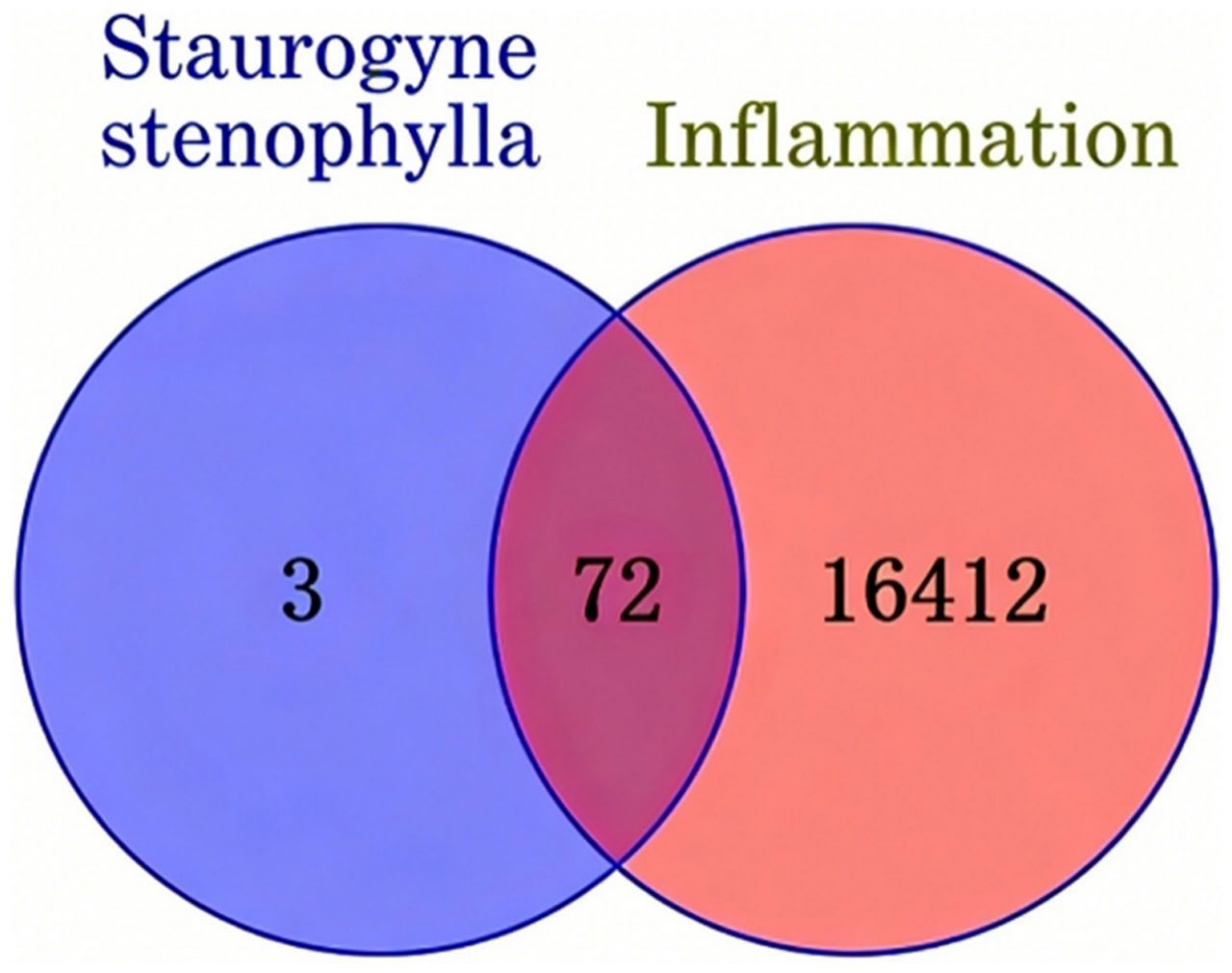
Venn diagram of *S. stenophylla* active component targets and inflammation targets.

Using inflammation-related keywords, a total of 16,484 inflammation-related targets were retrieved from the GeneCards and OMIM databases. These targets were matched with the 75 targets predicted from the active components. Ultimately, 72 potential intersecting targets were identified as key targets for the anti-inflammatory effects of *S. stenophylla* (Figure 4).

#### 3.2.4 Construction of the “drug-component-target-inflammation” network

**Figure 5:**
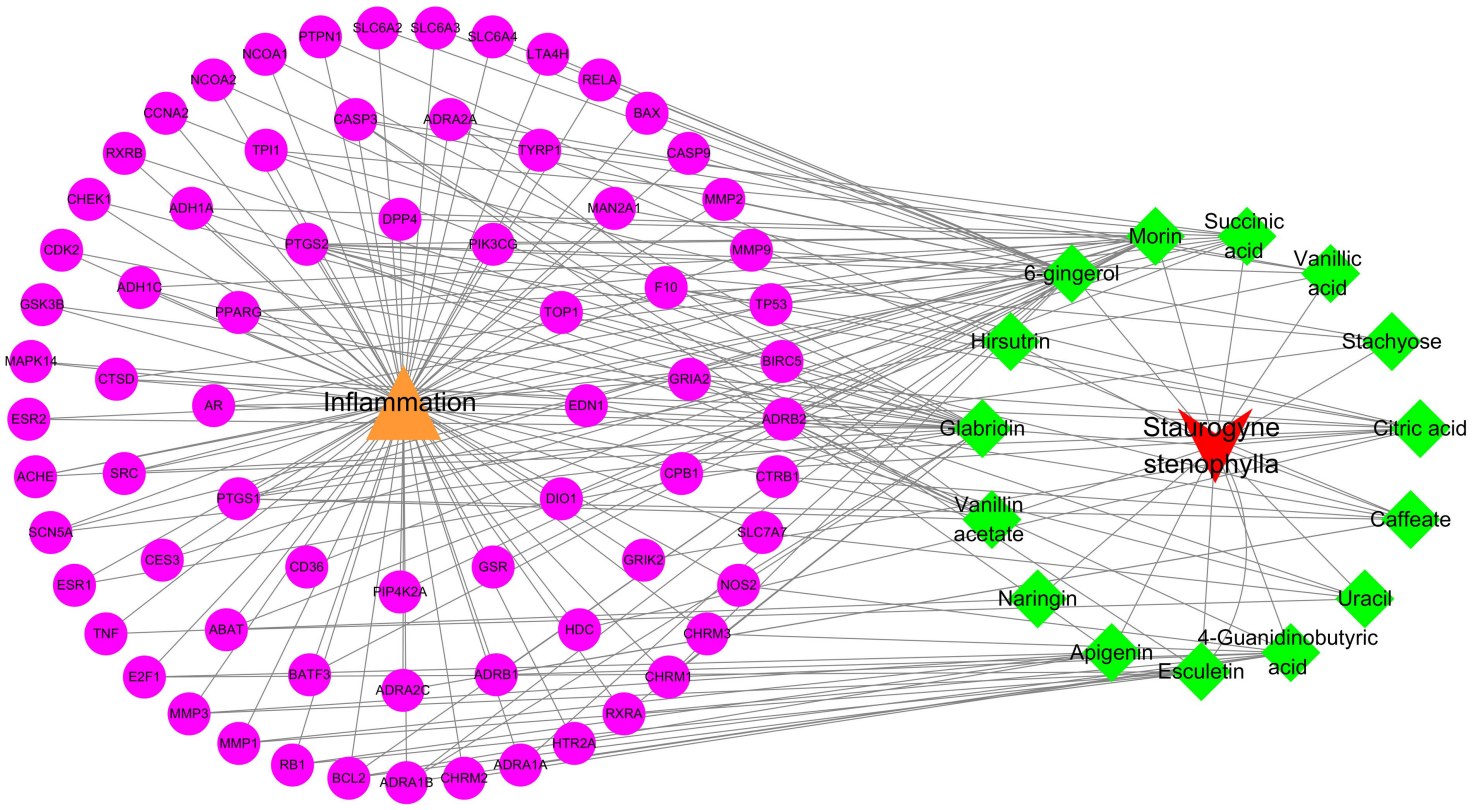
“Drug-Component-Target-Inflammation” network of *S. stenophylla*.

The 15 active components of *S. stenophylla* and the 72 potential anti-inflammatory intersecting targets were imported into Cytoscape software to construct and visualize the “Drug-Component-Target-Inflammation” network. The network comprises 1 herbal medicine, 1 disease node (inflammation), 88 nodes, 15 active components, and 207 edges (Figure 5). This finding highlights the characteristic therapeutic approach of herbal medicine, which exerts its effects through multi-component, multi-target, and multi-pathway mechanisms.

#### 3.2.5 PPI network construction and core target identification

**Figure 6:**
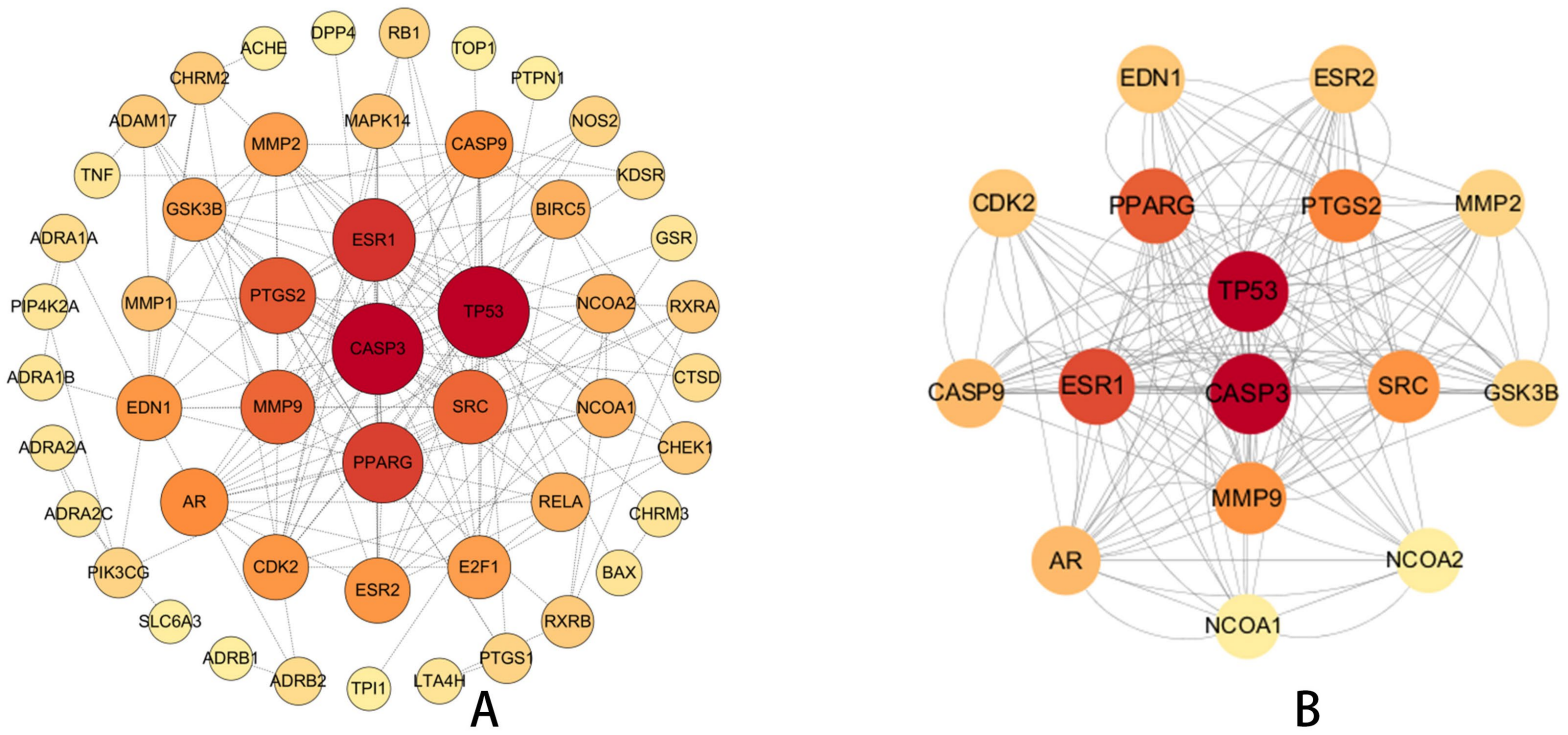
**(A)** PPI network and **(B)** core target network.

The 72 overlapping targets of *S. stenophylla* and inflammation were uploaded to the STRING database to analyze PPI relationships. Each node represents an interacting protein, while each line represents an interaction between targets; larger nodes indicate higher degree values. The results were visualized using Cytoscape software, generating a PPI network containing 58 nodes and 175 edges (Figure 6A). Furthermore, the topological parameters of the network nodes were calculated using the CytoNCA plugin, leading to the identification of 16 core targets (Figure 6B).

#### 3.2.6 GO function and KEGG pathway enrichment analysis

**Figure 7:**
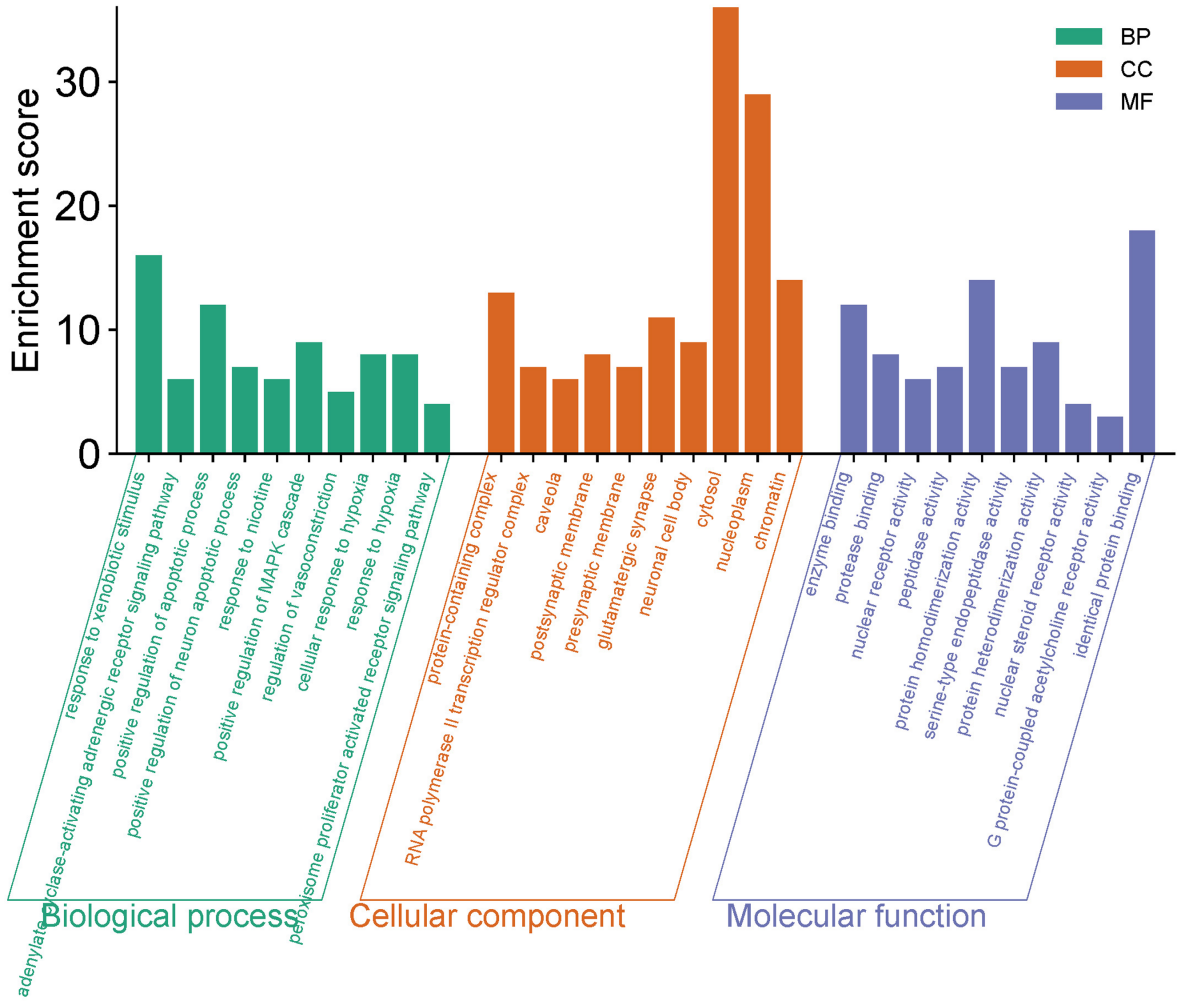
GO enrichment analysis (Top 10 terms for BP, CC, and MF).

GO functional enrichment and KEGG pathway enrichment analyses were performed on the 72 potential overlapping targets using the DAVID database, with *p* < 0.05 as the screening criterion. A total of 420 GO terms were identified. The GO analysis was categorized into three domains: biological process (BP), cellular component (CC), and molecular function (MF). The BP category was the most abundant, with 300 terms, mainly involving response to external biotic stimulus, positive regulation of apoptotic process, adenylate cyclase-activating adrenergic receptor signaling pathway, and positive regulation of MAPK cascade. The CC category contained 41 terms, primarily involving the plasma membrane, cytoplasm, and nucleus. The MF category included 79 terms, mainly focused on enzyme binding, protease binding, and nuclear steroid receptor activity. The top 10 terms for BP, CC, and MF, ranked by count, were visualized using a bar chart (Figure 7).

**Figure 8:**
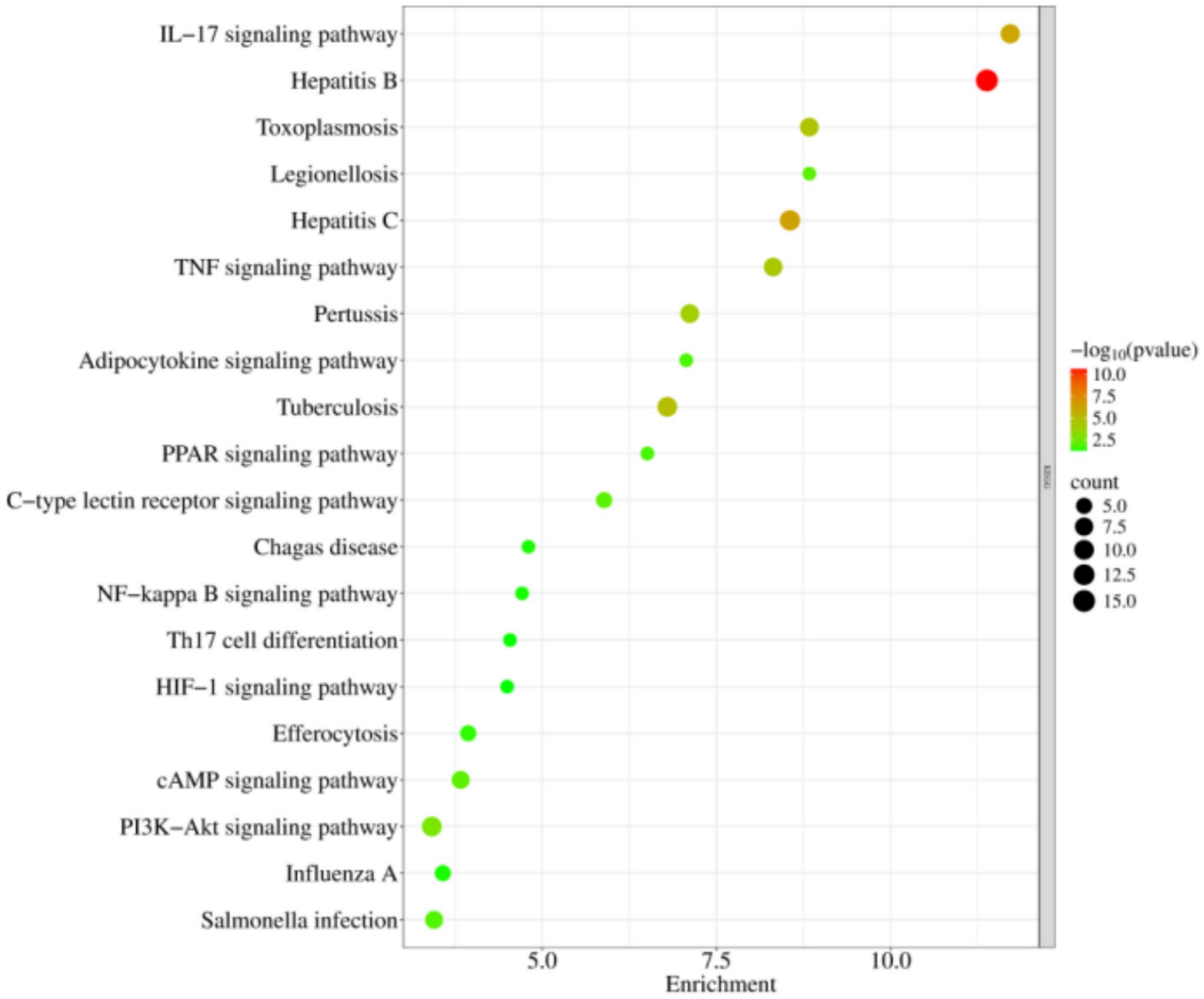
KEGG pathway enrichment analysis (Top 20 pathways).

KEGG pathway enrichment analysis yielded 99 enriched signaling pathways based on the criteria of *p* < 0.05 and Q < 0.05. The top 20 pathways were screened to construct a bubble chart. The results showed that the intersecting targets were primarily focused on pathways related to cytokine signaling, regulation of immune response, immune response induced by pathogen infection, and regulation of chronic inflammation (Figure 8).

#### 3.2.7 Molecular docking

**Figure 9:**
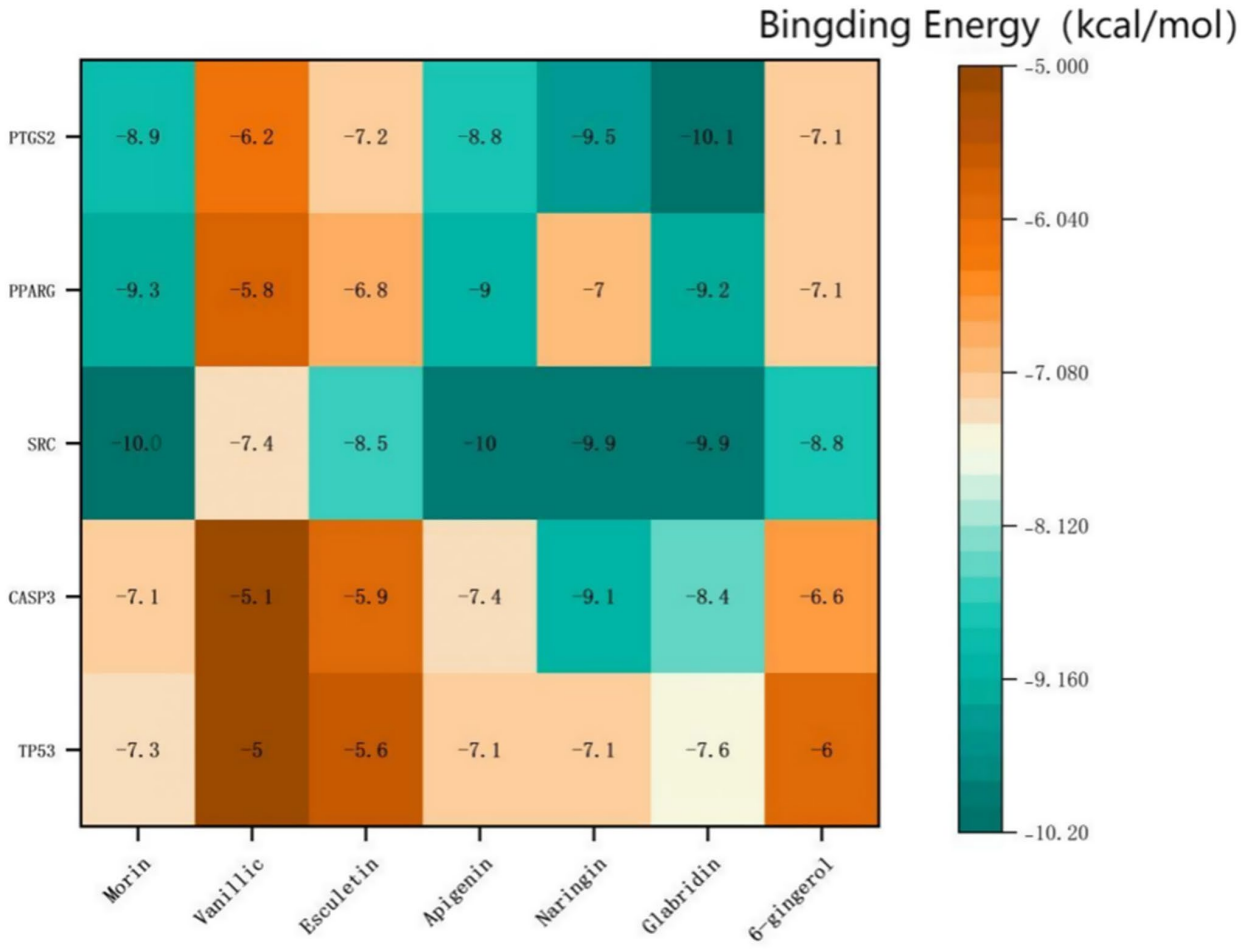
Heatmap of binding energies from molecular docking.

**Figure 10:**
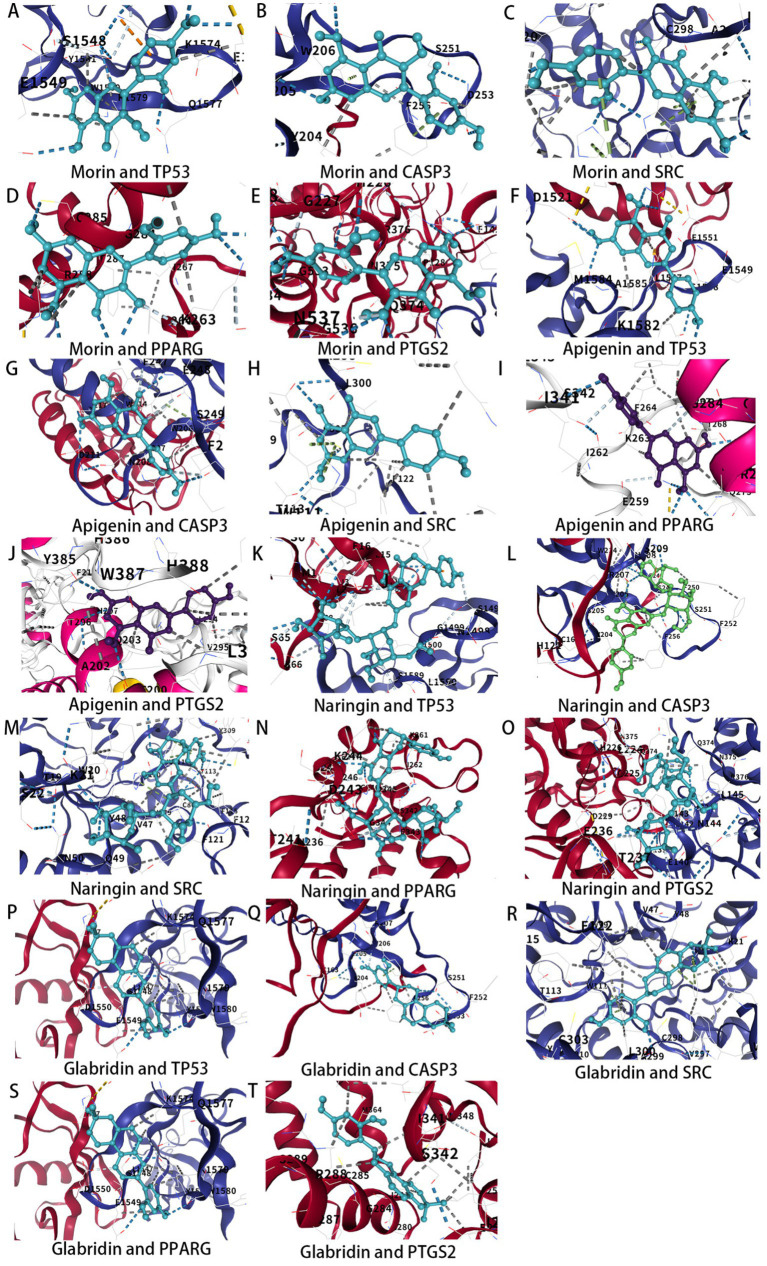
Visualization of molecular docking for *S. stenophylla* components and core inflammation targets. **(A)** Morin docked with TP53; **(B)** Morin docked with CASP3; **(C)** Morin docked with SRC; **(D)** Morin docked with PPARG; **(E)** Morin docked with PTGS2; **(F)** Apigenin docked with TP53; **(G)** Apigenin docked with CASP3; **(H)** Apigenin docked with SRC; **(I)** Apigenin docked with PPARG; **(J)** Apigenin docked with PTGS2; **(K)** Naringin docked with TP53; **(L)** Naringin docked with CASP3; **(M)** Naringin docked with SRC; **(N)** Naringin docked with PPARG; **(O)** Naringin docked with PTGS2; **(P)** Glabridin docked with TP53; **(Q)** Glabridin docked with CASP3; **(R)** Glabridin docked with SRC; **(S)** Glabridin docked with PPARG; **(T)** Glabridin docked with PTGS2.

From the 15 identified active components, 7 core active components (Morin, Vanillic acid, Esculetin, Apigenin, Naringin, Glabridin, and 6-Gingerol) were selected for molecular docking verification with 5 key core targets (TP53, CASP3, SRC, PPARG, and PTGS2). The minimum binding energy of each protein-ligand complex was calculated (Figure 9). In molecular docking, lower binding energy indicates better docking affinity and a more stable binding state between the ligand and receptor. Generally, a binding energy below zero suggests spontaneous binding, while a value ≤ − 6.0 kcal/mol indicates strong binding activity. In the heatmap, darker cyan indicates stronger binding activity. Subsequently, the complexes formed by Morin, Apigenin, Naringin, and Glabridin (binding energy < −7.0 kcal/mol) with their corresponding targets were selected for visualization (Figure 10).

#### 3.2.8 Molecular dynamics simulation

**Figure 11:**
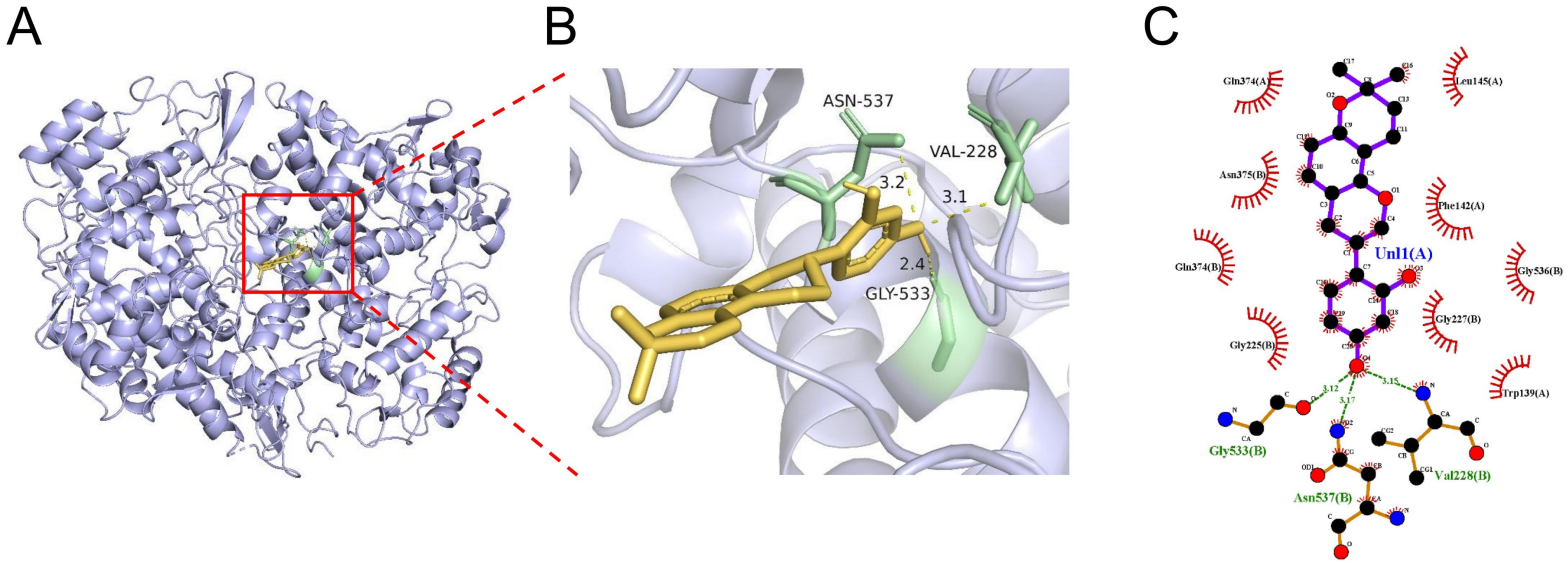
Molecular docking results of the PTGS2-Glabridin complex. (A) Overall view of the Glabridin-PTGS2 docking complex. (B) Detailed view of the binding interactions between Glabridin and key residues (ASN-537, VAL-228, GLY-533) in PTGS2. (C) 2D schematic diagram of the molecular interactions between Glabridin and PTGS2, showing hydrogen bonds and hydrophobic contacts.

**Figure 12:**
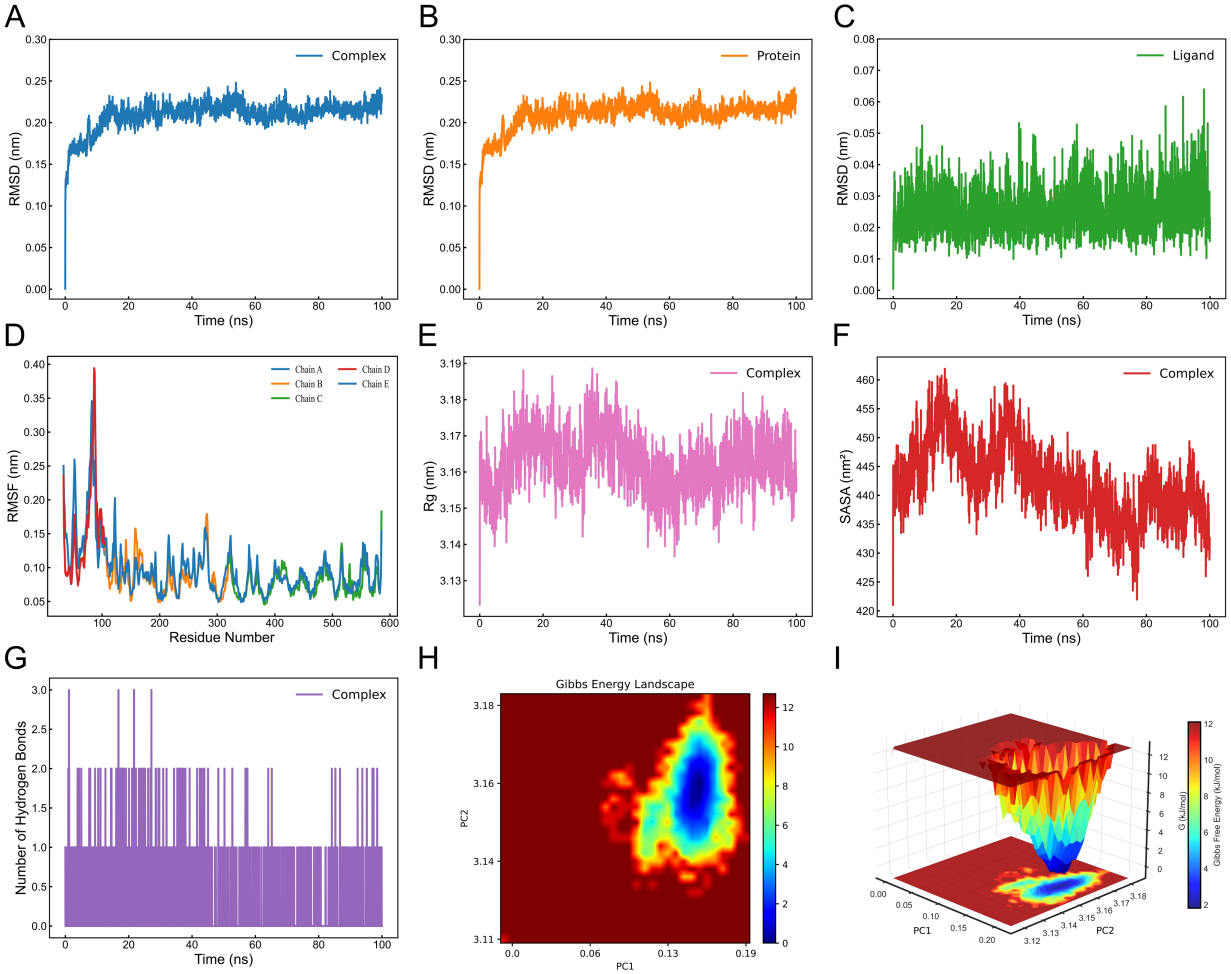
Molecular dynamics simulation results for the PTGS2-Glabridin complex. **(A–C)** RMSD plots of complex, protein backbone and ligand, respectively; **(D)** RMSF of residues for PTGS2 chains A–E; **(E)** Rg profile of the complex; **(F)** SASA fluctuation of the complex; **(G)** Time evolution of intermolecular hydrogen bonds; **(H, I)** 2D and 3D Gibbs free energy landscapes based on PCA.

Molecular dynamics (MD) simulation is a crucial technique for analyzing conformational changes and stability of receptor-ligand complexes. To investigate the binding stability of the PTGS2-Glabridin complex identified from molecular docking (Figures 11A–C), MD simulations were performed. As shown in Figures 12A–G, the complex gradually stabilized after 20 ns of simulation, indicating that the system had reached equilibrium. The curves for RMSD, RMSF, Rg, and SASA all exhibited favorable stability. Furthermore, the 2D and 3D free energy landscape (FEL) results (Figures 12H–I) revealed a distinct and deep energy basin for the PTGS2-Glabridin complex, demonstrating that the system formed a predominant stable conformation in this region.

## 4 Discussion

*Staurogyne stenophylla* has long been recognized in traditional Chinese folk medicine for its broad-spectrum anti-inflammatory properties, yet the specific molecular mechanisms underlying its anti-inflammatory effects remain poorly elucidated. Therefore, the present study characterized the potential bioactive components of *S. stenophylla* using UPLC-HRMS technology, initially identifying morin, vanillic acid, esculetin, apigenin, naringin, glabridin, and 6-gingerol as the core bioactive constituents. All seven core components were found to suppress the activation of NF-κB and MAPK signaling pathways, thereby attenuating the release of pro-inflammatory mediators. Mechanistically, morin and vanillic acid block the NF-κB pathway by inhibiting the phosphorylation of IκB kinase; simultaneously, they reduce the phosphorylation levels of MAPK family members (ERK, JNK, and p38), which constitutes one of the critical mechanisms mediating their anti-inflammatory activity (23–26). Esculetin exerts anti-inflammatory effects by directly targeting the key activation steps of the NF-κB and MAPK pathways, combined with its synergistic antioxidant activity (27, 28). Naringin modulates the NF-κB and MAPK pathways through two complementary strategies: inhibiting the upstream PI3K/Akt signaling cascade and inducing M2 polarization of macrophages (29, 30). Glabridin exerts its function via multi-targeted inhibition of the NF-κB and MAPK pathways at multiple nodes, as well as blocking their upstream cross-regulatory pathways (31, 32). In contrast, 6-gingerol indirectly suppresses these pathways by targeting downstream effector molecules and scavenging reactive oxygen species (ROS) (33). As a flavonoid compound ubiquitously distributed in various plants, apigenin binds to IRAK4 to block the activation of NF-κB and MAPK pathways (34); additionally, it downregulates the expression of inducible nitric oxide synthase (iNOS), thereby inhibiting nitric oxide (NO) synthesis (35). Notably, apart from apigenin, vanillic acid and glabridin also exhibit the ability to downregulate iNOS expression, leading to reduced NO production and subsequent suppression of inflammatory responses (36, 37). Furthermore, vanillic acid and 6-gingerol share a common “antioxidant-aided anti-inflammatory” mechanism: vanillic acid scavenges free radicals, while 6-gingerol eliminates ROS, both of which indirectly alleviate inflammation by mitigating oxidative stress (36, 38). Collectively, these core components were screened and validated using UPLC-HRMS technology. Their anti-inflammatory efficacy is achieved through a dual-mode action: directly inhibiting the activation of pro-inflammatory signaling pathways or downregulating the expression of effector molecules to reduce the release of inflammatory mediators, and indirectly enhancing anti-inflammatory effects via inherent antioxidant activity to alleviate oxidative stress. These findings collectively demonstrate that the identified core bioactive components play pivotal roles in mediating the anti-inflammatory effects of *S. stenophylla.*

In the present study, a preliminary investigation into the anti-inflammatory mechanisms of potential bioactive components from *S. stenophylla* was conducted based on a network pharmacology approach. Venn diagram analysis revealed a significant overlap between the targets of its potential bioactive components and those associated with inflammatory diseases, indicating that these bioactive components may participate in the regulation of inflammatory processes by acting on these shared intersecting targets. According to the PPI network analysis, SRC, PPARG, TP53, PTGS2, and CASP3 were identified as core anti-inflammatory targets. As an early key kinase in poultry inflammatory signal transduction, SRC serves as a crucial upstream switch for activating the NF-κB and MAPK pathways; inhibiting its expression can effectively block inflammatory responses at the source, which has been verified in poultry viral infection models (39, 40). As a nuclear receptor transcription factor, PPARG exerts extensive anti-inflammatory and immunomodulatory effects at the transcriptional level by antagonizing the transcriptional activity of NF-κB and modulating MAPK-related signaling, serving as an important target for regulating various inflammatory responses in livestock and poultry (41–43). TP53 acts as a dual-functional inflammatory regulator in livestock and poultry: it modulates the activity of NF-κB and MAPK pathways by balancing the expression of pro-inflammatory and anti-inflammatory factors (44, 45). PTGS2 is a key downstream effector enzyme in the inflammatory response of livestock and poultry. The activation of NF-κB and MAPK pathways can directly upregulate its transcription, thereby catalyzing the synthesis of pro-inflammatory mediators such as PGE2; conversely, inhibiting PTGS2 activity reduces their generation (46, 47). Furthermore, the NF-κB and MAPK pathways regulate CASP3 activation through transcriptional and post-translational mechanisms. Upon activation, CASP3 can eliminate overactivated inflammatory cells and inhibit NLRP3 inflammasome-mediated inflammatory amplification, constituting a key negative feedback axis for inflammation-apoptosis homeostasis (48, 49). Molecular docking results demonstrated that the binding energies between core components and core targets were all below −6 kcal/mol, indicating strong binding affinity. This suggests that the core components can stably bind to the above-mentioned anti-inflammatory core targets, and exert anti-inflammatory effects by regulating their mediated signaling pathways such as NF-κB and MAPK. The anti-inflammatory functions of these targets are consistent with the results of GO functional enrichment and KEGG pathway analysis, further confirming that the potential bioactive components of *S. stenophylla* exert anti-inflammatory effects by acting on shared targets of inflammation.

The NF-κB and MAPK signaling pathways constitute the core regulatory network of inflammatory responses in livestock and poultry, which are significantly activated in intestinal and respiratory inflammation triggered by lipopolysaccharide stress, mycotoxin damage, mycoplasma, and viral infections in poultry (46, 50), dominating the secretion of pro-inflammatory factors and the progression of inflammatory cascades. Combined with the network pharmacology prediction results of this study, the potential active components of *S. stenophylla* may exert anti-inflammatory effects by targeting pathways such as NF-κB/MAPK. Notably, in the interactions between core components and targets, the binding energy of PTGS2 with glabridin was as low as −10.1 kcal/mol. Combined with evidence from existing studies confirming that glabridin-mediated regulation of PTGS2 involves not only direct inhibition but also the intervention in complex upstream signaling pathways (51, 52), this finding is highly consistent with the aforementioned pathway regulatory logic. By simultaneously suppressing the activation of two major inflammatory signaling pathways (NF-κB and MAPK), glabridin potently downregulates the expression of downstream key effector enzymes (PTGS2/COX-2 and iNOS) and reduces the production of multiple pro-inflammatory cytokines. Additionally, it activates endogenous antioxidant pathways, thereby synergistically blocking the inflammatory process via a multi-level and multi-target mode of action (37, 53). To this end, the present study further performed molecular dynamics simulation on the PTGS2-glabridin complex. The results revealed a distinct deep energy basin in their binding conformation, indicating favorable binding stability. This outcome not only compensates for the limitation that static molecular docking fails to reflect the dynamic binding state under physiological conditions, but also clarifies the stable interaction mode between glabridin and PTGS2. It thus provides direct and reliable molecular mechanistic evidence for glabridin exerting anti-inflammatory effects via targeting PTGS2 and its upstream related pathways.

Based on the mechanistic predictions of this study and existing reports on the application of phytogenic feed additives in poultry, *S. stenophylla* extract possesses significant potential to be developed as an anti-inflammatory feed additive for livestock and poultry. With reference to the conventional application doses of similar plant extracts in poultry farming, the expected effective inclusion level is 0.1–0.5% (w/w), with a safety upper limit exceeding 1%; furthermore, LC–MS can be employed for the quantitative quality control of key active components to ensure batch stability of the extract, providing support for its large-scale production and farming application (54, 55). Nevertheless, certain limitations exist in the present study. The chemical constituents of *S. stenophylla* were only preliminarily characterized using non-targeted high-resolution mass spectrometry (annotated at MSI Level 2 without authentic standards), and the network pharmacology and molecular docking analyses could only predict potential anti-inflammatory mechanisms at a theoretical level, lacking functional experimental validation. Future research should be deepened and improved in two aspects: firstly, targeted analytical methods such as comparison with reference standards and standard curve-based quantification should be adopted to perform structural confirmation and quantitative analysis of taxonomically rare chemical constituents (e.g., glabridin), thereby enhancing the accuracy and reliability of the identification results; secondly, *in vitro* anti-inflammatory functional validation experiments should be conducted to clarify the anti-inflammatory effects of *S. stenophylla* extract and its key active components, comprehensively evaluating its application efficacy as a feed additive. These efforts will provide a reliable experimental basis for the resource development and scientific application of this resource in healthy livestock and poultry farming.

## 5 Conclusion

The core bioactive components of *S. stenophylla*, namely morin, vanillic acid, esculetin, apigenin, naringin, glabridin, and 6-gingerol, can specifically target key functional targets including SRC, PPARG, TP53, PTGS2, and CASP3. Among these interactions, the binding energy between PTGS2 and glabridin was the lowest, indicating superior binding stability and specificity of this component-target pair. Collectively, these core components exert anti-inflammatory effects primarily through the modulation of NF-κB and MAPK signaling pathways, thereby providing a theoretical basis for the development of *S. stenophylla*-derived products as feed additives for livestock and poultry.

## Data Availability

The original contributions presented in the study are publicly available. This data can be found here: All core experimental LC-MS data have been fully deposited in MetaboLights with accession number MTBLS14695, https://www.ebi.ac.uk/metabolights/MTBLS14695.

## Footnotes

1
https://www.tcmsp-e.com/#/home

2
https://www.uniprot.org/

3
https://auth.lifemapsc.com/

4
https://omim.org/

5
http://www.liuxiaoyuyuan.cn/

6
https://cn.string-db.org/

7
https://davidbioinformatics.nih.gov/

8
https://www.bioinformatics.com.cn/

9
https://pubchem.ncbi.nlm.nih.gov/

10
https://www.rcsb.org/
